# Cellular microRNA miR-181b Inhibits Replication of Mink Enteritis Virus by Repression of Non-Structural Protein 1 Translation

**DOI:** 10.1371/journal.pone.0081515

**Published:** 2013-12-11

**Authors:** Jia-zeng Sun, Jigui Wang, Daoli Yuan, Shuang Wang, Zhili Li, Bao Yi, Yaping Mao, Qiang Hou, Weiquan Liu

**Affiliations:** State Key Laboratory of Agrobiotechnology, Department of Biochemistry and Molecular Biology, College of Biological Sciences, China Agricultural University, Beijing, China; University of British Columbia, Canada

## Abstract

Mink enteritis virus (MEV) is one of the most important viral pathogens in the mink industry. Recent studies have showed that microRNAs (miRNAs), small noncoding RNAs of length ranging from 18–23 nucleotides (nt) participate in host-pathogen interaction networks; however, whether or not miRNAs are involved in MEV infection has not been reported. Our study revealed that miRNA miR-181b inhibited replication of MEV in the feline kidney (F81) cell line by targeting the MEV non-structural protein 1 (NS1) messenger RNA (mRNA) coding region, resulting in NS1 translational repression, while MEV infection reduced miR-181b expression. This is the first description of cellular miRNAs modulating MEV infection in F81 cells, providing further insight into the mechanisms of viral infection, and may be useful in development of naturally-occurring miRNAs antiviral strategies.

## Introduction

Mink enteritis virus (MEV) is one of the most important viral pathogens in the mink industry, resulting in huge economic losses in the worldwide. MEV, a subspecies of the feline parvovirus (FPV), contains a single-stranded negative sense DNA genome of about 5 kb, with 2 main open reading frames (ORFs) [1]. As do other parvoviruses, MEV causes a highly infectious acute disease and has a high rate of morbidity and mortality in mink [2]–[4]. Certain MEV vaccines have been used with some success to prevent further spread of the viral disease with significant decreases in morbidity and mortality [1], [5]–[8]. However, due to the genetic variability of the virus these vaccines are becoming increasingly inadequate and a simpler approach to controlling MEV infection would be advantageous.

miRNAs are endogenous small noncoding RNAs of length 18–23 nucleotides (nt), which play critical roles in many biological processes including cell proliferation, haematopoiesis and development of the nervous system [9]–[11]. miRNAs work by basically two modes [12]–[14]. In plants, they target mRNAs by precise or nearly precise complementary base pairing, and cleave target mRNAs directly [14]. In animals, being mostly imprecisely complementary to their mRNA targets, they often show translational repression and then lead to mRNA degradation [10], [15], [16]. Recent studies have also noted the role of miRNAs as modulators in host-pathogen interaction networks [17], [18]. Cellular miRNA hsa-miR-146a facilitates proliferation of Epstein-Barr (EB) virus by down regulation of an interferon-related gene [19]. Cellular hsa-miR-29a inhibits replication of human immunodeficiency virus (HIV) by targeting the viral Nef mRNA directly [20]. miR-323, miR-491 and miR-654 from both 293T and MDCK cells target the same region in H1N1 influenza virus PB1 mRNAs, thereby affecting the ability of the latter to replicate [21]. Virus-encoded miRNAs also play roles in viral infection. EB virus-derived miRNA miR-BART2 decreases replication of EB virus by targeting viral DNA polymerase BALF5 [22]. miR-BART22 helps EB virus evade the host immune response by reducing levels of EB virus latent membrane protein 2A (LMP2A) [23]. Human cytomegalovirus (HCMV)-encoding miR-US25-1c blocks the cell cycle at the G1/S phase by down regulating cyclin E2, BRCC3, EID1, MAPRE2, and CD147 to help the virus create a cellular environment conducive to DNA replication [24].

Results presented here show that cellular miR-181b in F81 cells inhibits replication of MEV by targeting its NS1 mRNA coding region resulting in NS1 translational repression, while MEV infection down regulates cellular miR-181b expression in F81 cells.

## Materials and Methods

### Animals and Ethics Statement

The mink were fed the same diet and euthanized according to local standards of animal welfare issues. All excised tissues were flash frozen in liquid nitrogen and stored at −80°C. Experiments involving animals were approved by the animal ethics committee of China Agricultural University with approval number XK320.

### Cell Culture and MEV Infection

F81 cells obtained from the American Type Culture Collection (ATCC) were cultured in MEM (Gibco, CA) containing 10% FBS (Hyclone, Logan, UT), and 1% penicillin-streptomycin (Gibco) at 37°C in a 5% CO_2_ atmosphere. MEV strain L was originally isolated from an infected farm animal, Liaoning province, China. The whole viral genome which is highly homologous with MEV strain Abashiri (GenBank accession, D00765.1) has been sequenced in our laboratory.

### Small RNA Ultrahigh throughput Sequencing and Analysis of Sequencing Data [25]

Three cultures of F81 cells in 6-well plates (Costar) were infected with MEV at an input multiplicity (MOI) of 1 pfu/cell. Three cultures of uninfected cells in 6-well plates were maintained as a control. Following the appearance of cytopathic effects (CPE) after infection, the triplicate cultures were pooled and RNA was then extracted by Trizol reagent (Invitrogen) and small RNAs were separated by PAGE. Bands corresponding to molecules of 18–30 nt were isolated and 10 µg aliquots were submitted to Solexa (now Illumina Inc.) for sequencing as cDNA libraries. Identical sequences in the infected and control samples were eliminated from the initial data set. The resulting sets of unique reads were mapped onto the feline genome [26], [27] using the program Short Oligonucleotide Analysis Package (SOAP) [28]. Perfectly matched reads were also mapped onto the miRNAs of six reference species (*Homo sapiens*, *Canis familiaris*, *Mus musculus*, *Rattus norvegicus*, *Bos taurus* and *Sus scrofa*) of the Sanger miRBase (Release 18) using Patscan [29] to identify homologs of known miRNAs.

### Prediction of miRNA Targets in MEV and Potential Target Conservation Analysis

RNAhybrid [30] (http://bibiserv.techfak.uni-bielefeld.de/rnahybrid/submission.html/) tools were used to predict miRNA targets in MEV mRNA following the rules of no mismatch and G/U complementarity in miRNA seed sequences. RegRNA [31] (http://vita.mbc.nctu.edu.tw/) tools were also used to predict regular RNAs on MEV mRNAs. For potential target conservation analysis, the target sequence was searched using BLAST (http://blast.ncbi.nlm.nih.gov/Blast.cgi). TargetScan tools were used to predict miRNA targets in known genes (http://www.targetscan.org/).

### Plasmid Construction

The luciferase expression vector pGL3-control (Promega) was used to construct miRNA candidate target analysis with pRL-TK (Promega) as control. The miR-181b candidate target segment of MEV, miR-181t, was amplified by PCR from the MEV genome and was directionally cloned into the 3′UTR of the luciferase gene in the pGL3-control vector, pGL3-181t. To facilitate cloning, an *Xba* I restriction site (underlined) was added to the 5′-(5′-GCTCTAGATCGGAAGTTGATAGTCTCGCC-3′) and 3′-(5′-GCTCTAGAGCCTTGATCTTTTCCCCATTC-3′) primers. To further ensure the miR-181b binding site in the NS1 gene indeed existed, a commercially synthesized (TaKaRa) seed mutant segment containing a tetranucleotide mutation was cloned into the pGL3-control vector to produce mut pGL3-181t.

For western blot assays, the coding region of the MEV NS1 protein gene fragment was amplified by PCR using the MEV genome as a template. For this cloning, *Bam*H I and *Age* I restriction sites (underlined) were added to the 5′-(5′-CGCGGATCCACCATGTCTGGCAACCAGTATACTG-3′) and the 3′-(5′-CGGCACCGGTATCCAAGTCGTCTCGAAAATCTTC-3′) primers respectively. For fusion with a His tag, the stop codon was deleted. The amplified fragments were then cloned into the *Bam*H I and *Age* I sites of pcDNA3.1/myc-His A vector (Invitrogen), generating pcDNA-NS1. The recombinant plasmid was sequenced (Shanghai Sangong Co.) to ensure the correct insertion.

### Construction of Mutant Plasmids

For further identification of the miR-181b binding site in the NS1 gene, the 3 nucleotides of the potential target site of pcDNA-NS1 and infectious clone vector pBM/rMEV were mutated using PCR. (The infectious clone vector pBM/rMEV was constructed using pBluescript II SK(+) Mutant in our laboratory.) Mutant plasmids were generated by PCR using PrimeSTAR MAX DNA Polymerase (TaKaRa), 50 ng of the parent vectors as templates and the complementary primers (forward primer: 5′-GTTTCTAAAAATATAGAACCAAACGAGTGCGTTTGGTTTATTC-3′ and reverse primer: 5′-GAATAAACCAAACGCACTCGTTTGGTTCTATATTTTTAGAAAC-3′) under the following conditions: 98°C for 3 min, followed by 30 cycles of 98°C for 10 s, 55°C for 15 s and 72°C for 90 s, followed by 72°C 10 min. The products were digested with 1 µl *Dpn*-1 for 1 h at 37°C to remove the parental DNA. The remaining DNA was used to transform competent DH5α cells, and the mutant plasmids were obtained and confirmed by sequencing (Shanghai Sangong Co.).

### miRNA Mimics and Inhibitors

The miR-21, miR-181b, miR-181c and miR-181d mimics and inhibitors, mut-1 miR-181b mimics and inhibitors (in which the tetranucleotide mutation was complementary to mut pGL3-181t) and mut-2 miR-181b mimics (in which the 3 nucleotides mutation was complementary to mut pcDNA-NS1 and mut pBM/rMEV) were synthesized by GenePharma, Shanghai. Mimics were double-stranded RNA oligos, while inhibitors were single-stranded. For control experiments, negative control mimics and inhibitors were also synthesized. All the inhibitors were modified by 2′-O-methyl and the sequences were listed as follows (underlined letters are mutated bases):

miR-21 mimics sense: 5′-UAGCUUAUCAGACUGAUGUUGA-3′

miR-21 mimics anti-sense: 5′AACAUCAGUCUGAUAAGCUAUU-3′

miR-181b mimics sense: 5′-AACAUUCAUUGCUGUCGGUGGGU-3′

miR-181b mimics anti-sense: 5′-CCACCGACAGCAAUGAAUGUUUU-3′

miR-181b inhibitors: 5′-ACCCACCGACAGCAAUGAAUGUU-3′

miR-181c mimics sense: 5′-AACAUUCAACCUGUCGGUGAGU-3′

miR-181c mimics anti-sense: 5′-UCACCGACAGGUUGAAUGUUUU-3′

miR-181d mimics sense: 5′-AACAUUCAUUGCUGUCGGUGGGU-3′

miR-181d mimics anti-sense: 5′-CCACCGACAGCAAUGAAUGUUUU-3′

miR-181d inhibitors: 5′-ACCCACCGACAGCAAUGAAUGUU-3′

Mut-1 miR-181b mimics sense: 5′-AACCGGUAUUGCUGUCGGUGGGU-3′

Mut-1 miR-181b mimics anti-sense: 5′-CCACCGACAGCAAUACCGGUUUU-3′

Mut-1 miR-181b inhibitors: 5′-ACCCACCGACAGCAAUACCGGUU-3′

Mut-2 miR-181b mimics sense: 5′-AGCACUCGUUGCUGUCGGUGGGU-3′

Mut-2 miR-181b mimics anti-sense: 5′-CCACCGACAGCAACGAGUGCUUU-3′

Negative control (NC) mimics sense: 5′-UUCUCCGAACGUGUCACGUTT-3′

Negative control (NC) mimics anti-sense: 5′-ACGUGACACGUUCGGAGAATT-3′

Negative control (NC) inhibitors: 5′-CAGUACUUUUGUGUAGUACAA-3′.

### Transfection

F81 cells were transfected with the plasmids and miRNA mimics or inhibitors using Lipofectamine 2000 transfection reagent (Invitrogen). To determine whether miRNAs play a direct role in repression of luciferase expression from pGL3-181t, 24-well microtitration plates (Costar) were seeded with F81 cells. Cells at 60–70% confluence were co-transfected with a mixture of pGL3-181t (0.1 µg/well) and pRL-TK vector (0.1 µg/well) together with mimics or inhibitors (10 pmol/well). The mut pGL3-181t vector and pRL-TK together with mimics or inhibitors (10 pmol/well) were co-transfected to verify accuracy of the seed sequence. NC mimics and NC inhibitors were used as negative controls. After 36 h, the cells were harvested for relative luciferase activity assay.

To determine whether miRNAs could down regulate the expression in pcDNA-NS1 or mut pcDNA-NS1, F81 cells, at a confluence of 60–70% in 6-well plates, were co-transfected with pcDNA-NS1 or mut pcDNA-NS1 (0.5 µg/well) with mimics or inhibitors (50 pmol/well). NC mimics and inhibitors were used as controls. At 36 h post-transfection, the cells were collected for NS1 mRNA qPCR assay and western blot analysis.

To determine the effects of the miRNAs on the replication of MEV or mut MEV, F81 cells, at a confluence of 60–70% in 24-well plates were transfected with mimics or inhibitors (10 pmol/well). After 12 h, the cells were dispersed with 0.25% trypsin and infected with MEV or mut MEV (MOI = 0.1). Viral replication was measured by virus titration (TCID_50_), CPE observation, indirect immunofluorescence assay and qPCR analysis.

### Luciferase Assays

Luciferase assays were performed using the dual-luciferase reporter assay system kit (Promega) according to the manufacturer’s protocol. Collected cells were washed once with cold phosphate-buffered saline (PBS). Passive lysis buffer (Promega: 100 µl) was then added to the cells. After 10 min, the supernatants were collected by centrifugation at 12,000 g for 30 s, and the luciferase activity was analyzed using a Modulus single-tube multimode reader (Promega). Relative luciferase expression was calculated as the expression of firefly luciferase (pGL3-control vector) divided by that of Renilla luciferase (pRL-TK).

### Real-time Quantitative PCR (qPCR) Analysis

To confirm that the miR-181b mimics and inhibitors function as expected, BCL-2 and transferrin receptor (TfR) of the F81 cells were chosen as positive and negative controls respectively. After transfection with miR-181b mimics and inhibitors, NC mimics and inhibitors as controls, total cellular RNA was extracted and digested with DNase I (Takara). Two µg total RNA was reverse transcribed using M-MLV reverse transcriptase (Promega) according to the manufacturer’s protocol. The level of β-actin mRNA was measured as a control. Primers for amplification were: β-actin, 5′-CGGGACCTGACGGACTACCT-3′ and 5′-GGCCATCTCCTGCTCAAAAT-3′; BCL-2, 5′-TGTGGAGAGCGTCAACCGAGAG-3′ and 5′-CAGGGACAGCCAGGAGAAATCA-3′; and TfR, 5′-ATGATTGGCTACTTGGGCTATTG-3′ and 5′-CCTGATGGTGCTGGTGAACTC-3′.

To determine the quantitative level of viral genomic DNA in F81 cells, total DNA was prepared and the concentration was measured. PCR amplication of a fragment of viral genomic DNA (5′-GCTTACGCTGCTTATCTTCGC-3′, 5′-TAATGTCCTATTTTCCCCCCC-3′) was carried out. To detect the level of expression of NS1 mRNA transcribed from pcDNA-NS1 vector, total RNA was prepared and digested with DNase I (Takara). Two µg total RNA was reverse transcribed using M-MLV reverse transcriptase (Promega) according to the manufacturer’s protocol. The level of β-actin mRNA was measured as a control. Primers were: β-actin, 5′-CGGGACCTGACGGACTACCT-3′ and 5′-GGCCATCTCCTGCTCAAAAT-3′; and NS1, 5′-CAAATGAAACCAGAAACCGT-3′ and 5′-TTTACTAACCAAGTCCCGCA-3′. To detect cellular expression level of miRNAs, total RNA was prepared, and 2 µg was polyadenylated using *E. coli* poly (A) polymerase according to the manufacturer’s protocol (Promega). The poly (A) reaction product was then reverse transcribed using M-MLV reverse transcriptase (Promega) and an adaptor primer [32] (5′-GCGAGCACAGAATTAATACGACTCACTATAGGTTTTTTTTTTTTVN-3′) according to the manufacturer’s protocol. PCR amplication of the miRNA cDNA was carried out using the appropriate miRNAs primers (listed in Table 1). For relative qPCR, the expression of U6 small RNA was measured as a control using the primers 5′-CTCGCTTCGGCAGCACA-3′ and 5′-AACGCTTCACGAATTTGCGT-3′. For absolute qPCR, serial 10-fold synthetic mimics dilutions were used to generate standard curves and the specific miRNAs were amplified to generate Ct values. Since it has been reported that a single mammalian cell contains 14.1 pg [33] or 25 pg [34] total RNA, miRNA copies/cell could be estimated. Cycling conditions for qPCR using FastSYBR Mixture (CWBIO) and the ViiA™ 7 real-time PCR System (Applied Biosystems) were 95°C for 20 s, followed by 35 cycles of 95°C for 3 s and 60°C for 30 s. The data were analyzed by the ΔΔCt method [35].

**Table 1:** qPCR primers for miRNAs.

miRNAs	Forward primer (5′–3′)	Reverse primer (5′–3′)
miR-181a	AACATTCAACGCTGTCGGTGAGTA	GCGAGCACAGAATTAATACGACTCAC
miR-181b	AACATTCATTGCTGTCGGTGGG	GCGAGCACAGAATTAATACGACTCAC
miR-181c	AACATTCAACCTGTCGGTGAGTAAA	GCGAGCACAGAATTAATACGACTCAC
miR-181d	AACATTCATTGTTGTCGGTGGGTA	GCGAGCACAGAATTAATACGACTCAC
miR-23a	ATCACATTGCCAGGGATTTCCA	GCGAGCACAGAATTAATACGACTCAC
miR-99a	AACCCGTAGATCCGATCTTGTGAA	GCGAGCACAGAATTAATACGACTCAC
miR-99b	CACCCGTAGAACCGACCTTGC	GCGAGCACAGAATTAATACGACTCAC
miR-301a	CGCAGTGCAATAGTATTGTCAAAGC	GCGAGCACAGAATTAATACGACTCAC
miR-26a	CGGTTCAAGTAATCCAGGATAGGCT	GCGAGCACAGAATTAATACGACTCAC
miR-26b	CGCGTTCAAGTAATTCAGGATAGGTA	GCGAGCACAGAATTAATACGACTCAC
miR-21	CGCGTAGCTTATCAGACTGATGTTG	GCGAGCACAGAATTAATACGACTCAC
miR-29a	CGTAGCACCATCTGAAATCGGTTA	GCGAGCACAGAATTAATACGACTCAC
miR-29b	CGTAGCACCATTTGAAATCAGTGTTA	GCGAGCACAGAATTAATACGACTCAC
miR-125b	TCCCTGAGACCCTAACTTGTGAAAA	GCGAGCACAGAATTAATACGACTCAC

### Western Blot Assay

Following transfection, the cells were collected and washed 3 times with cold PBS. The PBS was decanted, and then 100 µl RIPA lysis buffer (HX-BIO) and 0.5mM PSMF were added. After 30 min on ice and centrifugation at 12,000 g for 30 min, 20 µl supernatant was mixed with 20 µl 2×SDS sample buffer and boiled for 5 min. Samples were resolved on 10% SDS-PAGE gel and transferred to a nitrocellulose membrane (PALL Life Science). The membranes were blocked using 5% nonfat dry milk for 1 h, then incubated for 1 h at ambient temperature with purified primary mouse anti-His antibody and anti-β-actin antibody (MBL: 1∶2,500 and 1∶1,000 dilutions respectively) in nonfat milk. After 3 washes with Tris-buffered saline containing 0.05% Tween-20 (TBST), the membranes were incubated for 1 h at ambient temperature with the appropriate horseradish peroxidase-conjugated secondary antibody (MBL) at a 1∶5,000 dilution in TBST. Protein bands were visualized and analyzed using ImageJ software, with β-actin as a loading control.

### Indirect Immunofluorescence Assay

Following transfection with mimics or inhibitors, F81 cells were infected with MEV (MOI = 0.1). After 36 h, the cells were washed 3 times with PBS and fixed in cold fixative solution (acetone: methanol = 3∶1) for 10 min at 4°C followed by 3 washes with PBS and incubation for 1 h at 37°C with anti-MEV rabbit polyclonal antibody at 1∶100 (prepared in this laboratory). The cells were then washed 3 times with PBS, incubated with fluorescein isothiocyanate (FITC)-conjugated goat anti-rabbit IgG antibody (MBL: 1∶100 dilution) for 1 h at 37°C, washed a further 3 times with PBS, and observed by fluorescence microscopy.

### Flow Cytometry

F81 cell monolayers were detached with 0.25% trypsin and fixed in 4% paraformaldehyde, followed by 3 washes with PBS and incubation for 1 h at 37°C with anti-MEV rabbit polyclonal antibody at 1∶100. The cells were then washed 3 times with PBS, incubated with fluorescein isothiocyanate (FITC)-conjugated goat anti-rabbit IgG antibody (MBL: 1∶100 dilution) for 1 h at 37°C, washed a further 3 times with PBS and quantified by BD FACSCalibur flow cytometry. Nonspecific rabbit polyclonal antibody (iso) (prepared in this laboratory) was used as an isotype control. The data were analyzed using BD CellQuest software.

### RNA-induced Silencing Complex (RISC) Co-immunoprecipitation Assay

Human Argonaute 2 (Ago2) antibody (Abnova) was bound to protein A/G-Agarose (Abmart) in PBS for 30 min at 4°C. F81 cells were harvested, washed and lysed with RIPA lysis buffer (HX-BIO) and PSMF for 30 min on ice, then centrifuged at 12,000 g for 30 min to sediment particulates. The supernatant was then incubated with the Ago2/agarose for 4 h at 4°C. Incubation with normal mouse IgG (MBL) provided a negative control. RNA binding to the Ago2 protein was extracted with Trizol reagent (Invitrogen), then reverse transcribed into cDNA. NS1 and miR-181b were quantified by qPCR analysis, using β-actin and U6 small RNA as internal controls.

### Virus Titer Assay

Virus titration (TCID_50_ assay) was carried out in 96-well microtitration plates (Costar) seeded with F81 cells (10^5^ cells/well). Serial 10-fold virus dilutions in MEM (100 µl) were made in triplicate and the plates were incubated in 5% CO_2_ at 37°C. Wells were examined microscopically and endpoints were taken as the inverse of the highest dilution showing CPE. Titers were calculated by the Reed-Münch method.

### Statistical Analysis

Some data were processed with the appropriate statistical analysis described in figure legends.

## Results

### Screening of miRNAs Targeting MEV mRNAs and Confirmation of the Function of miR-181b Mimics and Inhibitors

**Figure 1:**
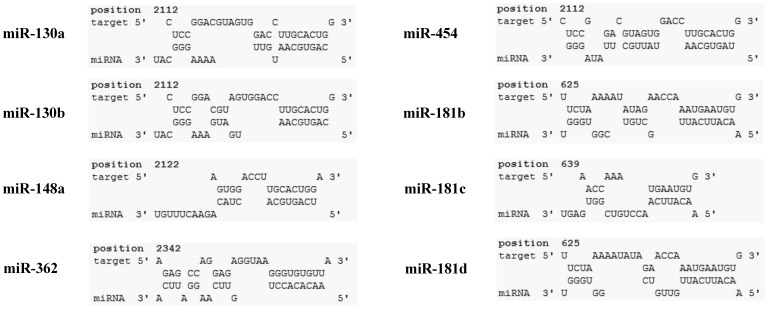
Screening of miRNAs that changed more than 2-fold on MEV infection targeting MEV mRNAs. RNAhybrid and RegRNA tools were used to predict miRNA target sites on MEV mRNAs following the rules of no mismatch and G/U complementary in miRNA seed sequences.

To identify potential miRNAs directly targeting MEV mRNAs, small RNA ultrahigh throughput sequencing was performed (Solexa) on uninfected F81 cells and following MEV (MOI = 1) infection as described in Materials and Methods. The resulting data was screened for miRNAs with more than a 2-fold change following MEV infection, and RNAhybrid [30] and RegRNA [31] tools were used to predict miRNA target sites on MEV mRNAs. Results indicated that 8 of such miRNAs might target MEV mRNAs (Figure 1). Among them, miR-181b showed comparatively the highest level expression (Table 2). To test whether endogenous (cellular) miR-181b was expressed at a high enough level (>100 copies/cell [34], [36]) to have any function, absolute qPCR analysis was performed. Results showed that the expression level of miR-181b was almost 800 copies/cell (>100 copies/cell) and more than that of miR-181c and miR-181d (Figure S1 *A*) Prediction results also showed that miR-181b might target MEV NS1 at the 9 nucleotide sequence coding region (334–342) of NS1 mRNA (Figure 1). This potential target site and the two flanking sequences are identical in all the known isolates of MEV, canine parvovirus (CPV) and feline panleukopenia virus (FPV) including MEV strain L (Table 3).

**Table 2:** Expression level of the predicted miRNAs that changed more than 2-fold on MEV infection of the two libraries.

miRNAs	Rds num^a^ in mockinfected cells	Rds num in MEVinfected cells	TPM^b^ in mockinfected cells	TPM in MEVinfected cells
miR-130a	72	332	12.67	59.23
miR-130b	264	584	46.44	104.18
miR-148a	510	163	89.72	29.08
miR-362	857	149	150.77	26.58
miR-454	76	1	13.37	0.18
miR-181b	3305	1393	581.44	248.50
miR-181c	1	2	0.18	0.36
miR-181d	386	142	67.91	25.33

^a^ Rds mum: Reads number.

^b^ TPM: Tags per million.

**Table 3:** The conserved binding sites of miR-181b in the NS1 gene of pavovirus strains^a^.

Virus strains	Sequence^e^
MEV/L^b^	…………………………aaaatgtctt tttgaagtct ttgtttctaa aaatatagaa cca***aatgaat gt***gtttggtt…………………………………..
MEV/LN-10	…………………………aaaatgtctt tttgaagtct ttgtttctaa aaatatagaa cca***aatgaat gt***gtttggtt…………………………………..
MEV/MEVB	…………………………aaaatgtctt tttgaagtct ttgtttctaa aaatatagaa cca***aatgaat gt***gtttggtt…………………………………..
MEV/Jlin/2010	…………………………aaaatgtctt tttgaagtct ttgtttctaa aaatatagaa cca***aatgaat gt***gtttggtt…………………………………..
MEV/Abashiri	…………………………aaaatgtctt tttgaagtct ttgtttctaa aaatatagaa cca***aatgaat gt***gtttggtt…………………………………..
CPV^c^	…………………………aaaatgtctt tttgaagtct ttgtttctaa aaatatagaa cca***aatgaat gt***gtttggtt…………………………………..
FPV^d^	…………………………aaaatgtctt tttgaagtct ttgtttctaa aaatatagaa cca***aatgaat gt***gtttggtt…………………………………..

^a^ Collected from NCBI 17 December 2012 and our lab sequencing.

^b^ The whole genome sequence of MEV in our lab is unpublished.

^c^ All the isolates of canine parvovirus from NCBI.

^d^ All the isolates of feline panleukopenia virus from NCBI.

^e^ Bold and italic sequences indicate conserved binding sites.

For verification, miRNAs mimics and inhibitors were synthesized. To confirm that they functioned as expected in F81 cells, mimics and inhibitors of miR-181b were selected and its known target, antiapoptotic gene BCL-2 [37], [38] was chosen as a positive control. The miR-181b target site (white box; Figure S1 *B*) in the 3′UTR of BCL-2 is conserved in different species, including cat (black box; Figure S1 *B*), with transferrin receptor gene (TfR) as a negative control. The analysis of TfR was carried out using TargetScan tools (data not shown). Results showed that, compared with TfR, BCL-2 expression level was significantly down regulated by miR-181b mimics, while the inhibitors restored the function of the endogenous miR-181b (Figure S1 *C*).

### Cellular Endogenous miR-181b Inhibits MEV Production

**Figure 2:**
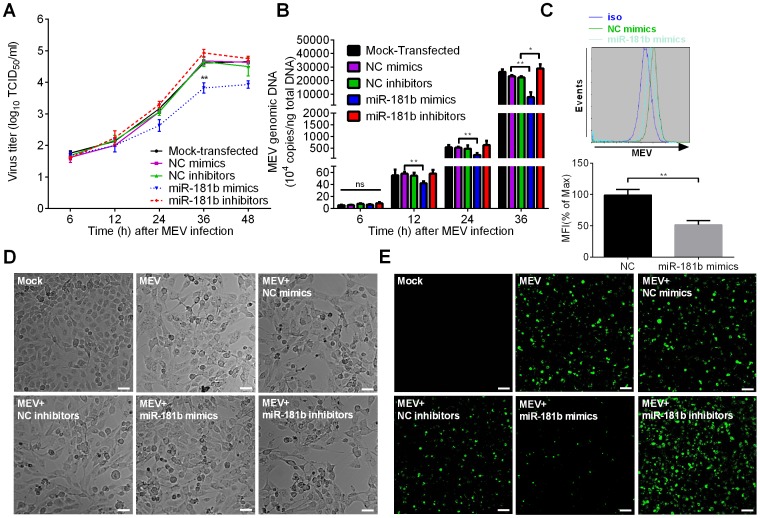
Cellular endogenous miR-181b inhibits MEV production. (**A**) TCID_50_ values were used to assess effects of miR-181b mimics and inhibitors on viral growth curves in F81 cells. NC mimics and inhibitors were used as controls. F81 cells were transfected with mimics or inhibitors for 12 h, and infected with MEV at an MOI of 0.1. Cells were collected at the indicated times and assayed. (**B**) qPCR was used to assess the effects of miR-181b mimics and inhibitors of MEV genomic DNA at the indicated times from **A)**. The experiments were performed as in **A)**. (**C**) F81 cells upon infection with MEV from **A)** were detected through flow cytometric analysis at 36 h post MEV infection, with nonspecific rabbit polyclonal antibody (iso) as an isotype control. (**D**) F81 cells from **A)** were observed for development of CPE by bright-field microscopy at 36 h post MEV infection. White scale bar: 50 µm. (**E**) F81 cells upon infection with MEV from **A)** were also observed by fluorescence microscopy through indirect immunofluorescence assay at 36 h post MEV infection. White scale bar: 50 µm. Data are representative of 3 independent experiments (mean ± SD). Statistical significance was analyzed by Student’s *t* test; * *P*<0.05; ** *P*<0.01; ns, not significant.

To investigate whether miR-181b could attenuate MEV replication, miR-181b mimics or inhibitors were used to transfect F81 cells before MEV infection, with the corresponding NC mimics or inhibitors as controls. At the indicated times, virus titer and genomic DNA levels were examined. As anticipated, miR-181b mimics inhibited viral growth and this was most evident 36 h post MEV infection, at which time the virus titer was significantly suppressed almost 8-fold by miR-181b mimics, while the inhibitors tended to prevent the inhibitory activity of the endogenous miR-181b on viral growth but the effect was very minor and not significant (Figure 2*A*). From 12 h post MEV infection, the level of viral genomic DNA was significantly down regulated by miR-181b mimics: by 36 h inhibition was almost 3-fold, while the miR-181b inhibitors did not show any effect at earlier timepoints of MEV infection but at 36 h they blocked endogenous miR-181b function and restored the viral growth rate significantly. By 36 h post infection, the level of the genomic DNA had increased 25% as compared with transfection by NC inhibitors (Figure 2*B*). Since all members of the same miRNA family share the same targets [39], we reasoned that the other two members (miR-181c and miR-181d) of the miR-181 family predicted from sequencing data would inhibit MEV replication. Results from virus titration and viral genomic DNA quantification showed that miR-181d mimics, like miR-181b mimics, could inhibit MEV growth, but restoration of the endogenous miR-181d function by miR-181d inhibitors was not detected (Figure S2 *A, B*). Surprisingly, miR-181c mimics did not affect MEV replication (Figure S2 *D, E*). Since at 36 h post MEV infection the effect of miR-181b was the most apparent, flow cytometry, CPE and MEV indirect immunofluorescence assays were also carried out at that time. Flow cytometry showed that, compared with transfection with NC mimics, miR-181b mimics significantly decreased the proportion of MEV infected cells by almost half (Figure 2*C*). Microscopically, miR-181b mimics significantly reduced MEV-induced CPE in F81 cells in comparison with NC mimics, showing more closely growing, more viable intact and fewer irregular dead cells, while miR-181b inhibitors increased CPE (Figure 2*D*). MEV indirect immunofluorescence assay also showed that miR-181b mimics decreased, and miR-181b inhibitors increased, the quantity of fluorescence (Figure 2*E*). Altogether, these results clearly show that cellular endogenous miR-181b has a negative effect on MEV replication.

### Cellular miR-181b Directly Targets MEV NS1 mRNA Coding Region

**Figure 3:**
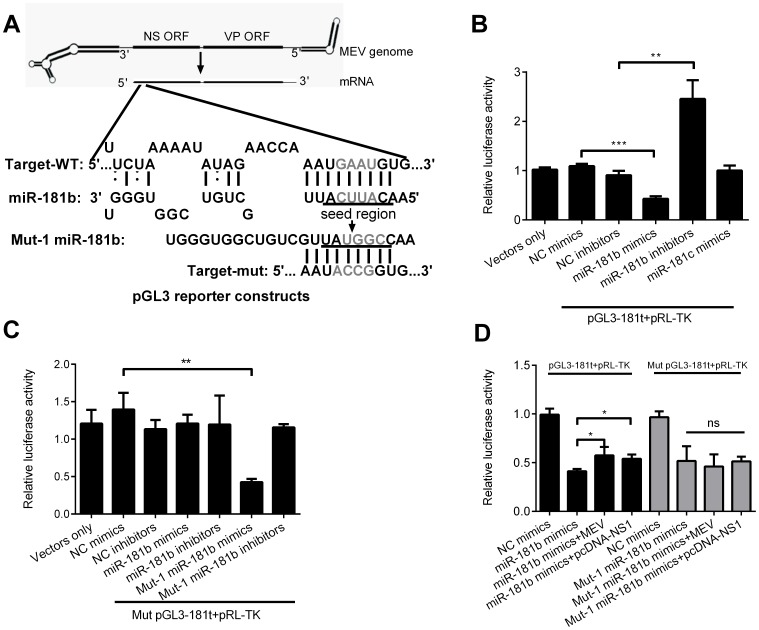
Cellular miR-181b directly targets MEV NS1 mRNA coding region. (**A**) Schematic layout of the MEV genome DNA, NS1 mRNA and presumptive target of miR-181b via the 9 sequential complementary nucleotides as indicated. The target and tetranucleotide mutant target segment was cloned into pGL3-control reporter vector. (**B**) Luciferase activity of lysates of F81 cells after 36 h co-transfection with pGL3-181t, pRL-TK and miR-181b or miR-181c mimics or inhibitors. NC mimics and inhibitors were used as controls. (**C**) As in **B)**, except that mut pGL3-181t, mut-1 miR-181b mimics and inhibitors were used for transfection. (**D**) Luciferase activity of lysates of F81 cells infected with MEV at an MOI of 0.1 or transfected with pcDNA-NS1 (0.1 µg/well), and co-transfected with reporter constructs (0.1 µg/well) and miRNA mimics (10 pmol/well) in 24-well plates. Data are from 3 independent experiments (mean ± SD). Statistical significance was analyzed by Student’s *t* test; *P<0.05; **P<0.01; ***P<0.001; ns, not significant.

To confirm that miR-181b could directly target region 334-342 of NS1 mRNA and show inhibitory activity, a reporter vector pGL3-181t containing the potential target segment in the 3′UTR of the luciferase gene was constructed (Figure 3*A*). After co-transfection of F81 cells with pGL3-181t and miR-181b mimics or inhibitors, the relative luciferase activity was measured. As predicted, miR-181b significantly inhibited the relative luciferase activity (by more than half) in comparison with NC mimics, while the inhibitors increased it (more than 2-fold) (Figure 3*B*). To provide further evidence that miR-181c did not act like miR-181b, the above experiment was repeated using miR-181c mimics. As previously shown, miR-181c did not affect the relative luciferase activity (Figure 3*B*). To further ascertain the necessary function of complementary seed sequence, a seed reporter vector, mut pGL3-181t, with a mutated tetranucleotide was constructed and the corresponding mut-1 miR-181b mimics and inhibitors were synthesized (Figure 3*A*). After co-transfection of F81 cells, as expected, miR-181b mimics and inhibitors did not affect the relative expression activity of mut pGL3-181t, but mut-1 miR-181b mimics did down regulate it by more than 2-fold (Figure 3*C*). To test whether MEV and the NS1 expression vector pcDNA-NS1 could interact with miR-181b, thereby competing with the reporter vectors, the treated cells were infected with MEV following co-transfection with the luciferase reporter vector and miRNA mimics. In addition, cells were co-transfected with the luciferase reporter vector, miRNA mimics and pcDNA-NS1. Results showed that both MEV and pcDNA-NS1 restored the inhibition of the relative expression activity of pGL3-181t vector by miR-181b mimics but did not contribute to the relative expression activity of mut pGL3-181t vector by mut-1 miR-181b mimics (Figure 3*D*). Although the miR-181b target site in the 3′ UTR of the luciferase gene is not representative of its position in the NS1 coding region, these results indicate that cellular miR-181b can directly target MEV NS1 mRNA coding region through a 9 nucleotide complementary seed.

### Cellular miR-181b Inhibits MEV NS1 Expression Resulting in Translational Repression through Targeting the Predicted Site of MEV NS1 mRNA

**Figure 4:**
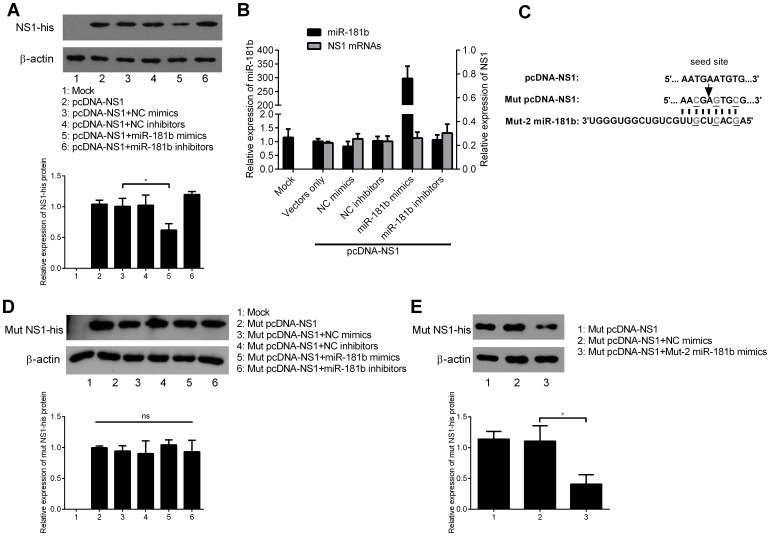
Cellular miR-181b inhibits MEV NS1 expression resulting in translational repression through targeting the predicted site of MEV NS1 mRNA. (**A**) Western blot assay of lysates of F81 cells after 36 h co-transfection with pcDNA-NS1 and either mimics or inhibitors. (**B**) qPCR was used to assess effects of miR-181b on the relative expression of NS1 in pcDNA-NS1 with β-actin as an internal control. The experiments were performed as in **A)**. (**C**) Schematic layout of mut pcDNA-NS1 vector. The nucleotide sequence of the presumptive target, but not the amino acid sequence, of the NS1 gene was altered. Altered nucleotides are underlined. The corresponding mut-2 miR-181b was synthesized. (**D**) Western blot assay was performed as in **A)** except that mut pcDNA-NS1 was used instead of pcDNA-NS1. (**E**) Western blot assay of F81 cells after 36 h co-transfection with mut pcDNA-NS1 and mut-2 miR-181b mimics with NC mimics as control. Data are from 3 independent experiments (mean ± SD). Statistical significance was analyzed by Student’s *t* test; *P<0.05; ns, not significant.

To elucidate the mechanism of inhibition of MEV NS1 expression by miR-181b, the NS1 coding region was cloned into pcDNA3.1/myc-His A vector, generating an NS1 carbon terminal fusion with a His tag expression vector (pcDNA-NS1). F81 cells were co-transfected with pcDNA-NS1 and miR-181b mimics or inhibitors, with NC mimics or inhibitors as controls. At 36 h post-transfection, RIPA lysis buffer (HX-BIO) was added and the protein lysates were harvested and subjected to western blot assay. Results showed that miR-181b significantly decreased NS1 protein levels by almost 40%, and miR-181b inhibitors enhanced these by about 20% (Figure 4*A*). However, NS1 mRNA, as measured by qPCR assay, was unaffected by either miR-181b mimics or inhibitors at 36 h post-transfection (Figure 4*B*). Altogether, these results demonstrate that cellular miR-181b inhibits MEV NS1 expression resulting in translational repression. To further confirm the significance of target sequence in decreased NS1 expression, mut pcDNA3.1-NS1 was constructed. The nucleotide sequence of the target, but not the amino acid sequence, of the NS1 gene was altered and the mut-2 miR-181b mimics (in which the 3-nucleotide mutation was complementary to mut pcDNA-NS1 and mut pBM/rMEV) were synthesized (Figure 4*C*). To check whether miR-181b could affect mut NS1 expression, mut pcDNA3.1-NS1 and miR-181b mimics or inhibitors were used to co-transfect F81 cells, using NC mimics and inhibitors as the respective controls. At 36 h post-transfection, RIPA lysis buffer-extracted protein lysates were harvested and a western blot assay performed. Results showed that miR-181b could not affect mut NS1 protein translation (Figure 4*D*). To test if mut-2 miR-181b mimics could restore the inhibition of mut NS1 expression, mut pcDNA3.1-NS1 and mut-2 miR-181b mimics were used to co-transfect F81 cells, with NC mimics as a control. Results showed that mut NS1 expression level was significantly down regulated by more than 50% by the mut-2 miR-181b mimics in comparison with NC mimics. miR-181c and miR-181d were also tested for their ability to attenuate NS1 protein expression. As anticipated, miR-181d mimics significantly down regulated NS1 expression but miR-181c did not (Figure S2 *C, F*). Altogether, these results demonstrate that cellular miR-181b inhibits MEV NS1 expression resulting in translational repression by targeting the predicted site of MEV NS1 mRNA.

### Cellular miR-181b Inhibits MEV Replication through Targeting the Predicted Site of MEV NS1 mRNA

**Figure 5:**
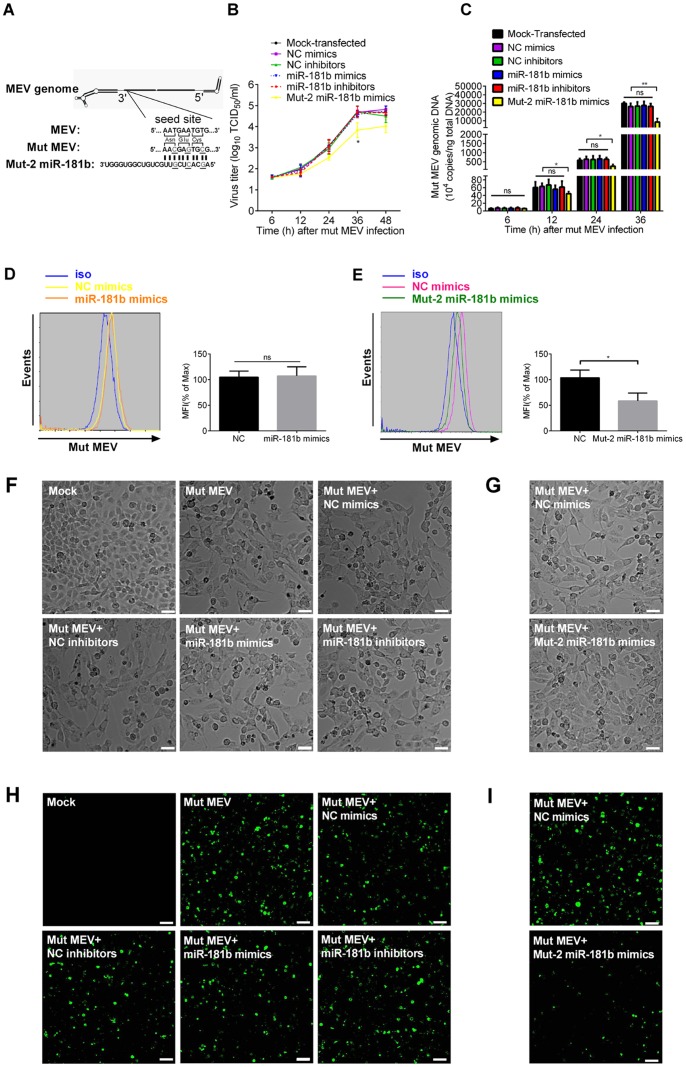
Cellular miR-181b inhibits MEV replication through targeting the predicted site of MEV NS1 mRNA. (**A**) Schematic layout of mut pMEV. The nucleotide sequence of the presumptive target, but not the amino acid sequence, of the NS1 gene was altered. Altered nucleotides are underlined. The corresponding mut-2 miR-181b was synthesized. (**B**) TCID_50_ values were used to assess effects of miR-181b mimics and inhibitors or mut-2 miR-181b mimics on viral growth curves of mut MEV in F81 cells. NC mimics and inhibitors were used as controls. F81 cells were transfected with mimics or inhibitors for 12 h and then infected with mut MEV (MOI = 0.1). Cells were collected at the indicated times and titrated. (**C**) qPCR was used to assess the effects of miR-181b mimics and inhibitors or mut-2 miR-181b mimics of mut MEV genomic DNA at the indicated times from **B)**. (**D, E**) F81 cells upon infection with mut MEV from **B)** were detected by flow cytometric analysis at 36 h post MEV infection, with nonspecific rabbit polyclonal antibody (iso) as an isotype control. (**F, G**) F81 cells from **B)** were observed for development of CPE by bright-field microscopy at 36 h post MEV infection. White scale bar: 50 µm. (**H, I**) F81 cells upon infection with mut MEV from **B)** were also observed by fluorescence microscopy using an indirect immunofluorescence assay. White scale bar: 50 µm. Data are from 3 independent experiments (mean ± SD). Statistical significance was analyzed by Student’s *t* test; * *P*<0.05; ** *P*<0.01; ns, not significant.

To further confirm the significance of target sequence in virus production, mut pBM/rMEV was constructed. The nucleotide, but not the amino acid, sequence of the target was altered (Figure 5*A*). To investigate whether miR-181b could inhibit mut MEV replication, miR-181b or mut-2 miR-181b mimics or miR-181b inhibitors were used to transfect. F81 cells before mut MEV infection, with the corresponding NC mimics and inhibitors as controls. At the indicated times, virus titers and the quantity of viral genomic DNA were measured. As predicted, results showed that miR-181b did not alter growth of mut MEV, while the corresponding mut-2 miR-181b mimics significantly down regulated mut MEV growth reaching a maximum at 36 h following mut MEV infection (The mut MEV virus titer and genomic DNA level were respectively reduced approximately 7-fold and 3-fold) (Figure 5*B, C*). To further validate the results above, at post 36 h mut MEV infection, flow cytometry, CPE and MEV indirect immunofluorescence assays were also performed. Flow cytometry showed that miR-181b had no effect on the quantity of the mut MEV infected cells whereas, compared with transfection with NC mimics, mut-2 miR-181b mimics significantly decreased it by almost 2-fold (Figure 5*D, E*). miR-181b did not affect the extent of mut MEV-induced CPE in F81 cells, but mut-2 miR-181b mimics reduced this by comparison with NC mimics, showing more closely growing, more viable intact and fewer irregular dead cells (Figure 5*F, G*). At the same time, MEV indirect immunofluorescence assay also showed that mut-2 miR-181b but not miR-181b mimics decreased the quantity of fluorescence (Figure 5*H, I*). Altogether, these results further establish that the 9 nucleotide target sequence of NS1 mRNA is of great importance for the action of miR-181b on MEV replication.

### Cellular miR-181b Physically Binds to MEV NS1 mRNA in the RISC

**Figure 6:**
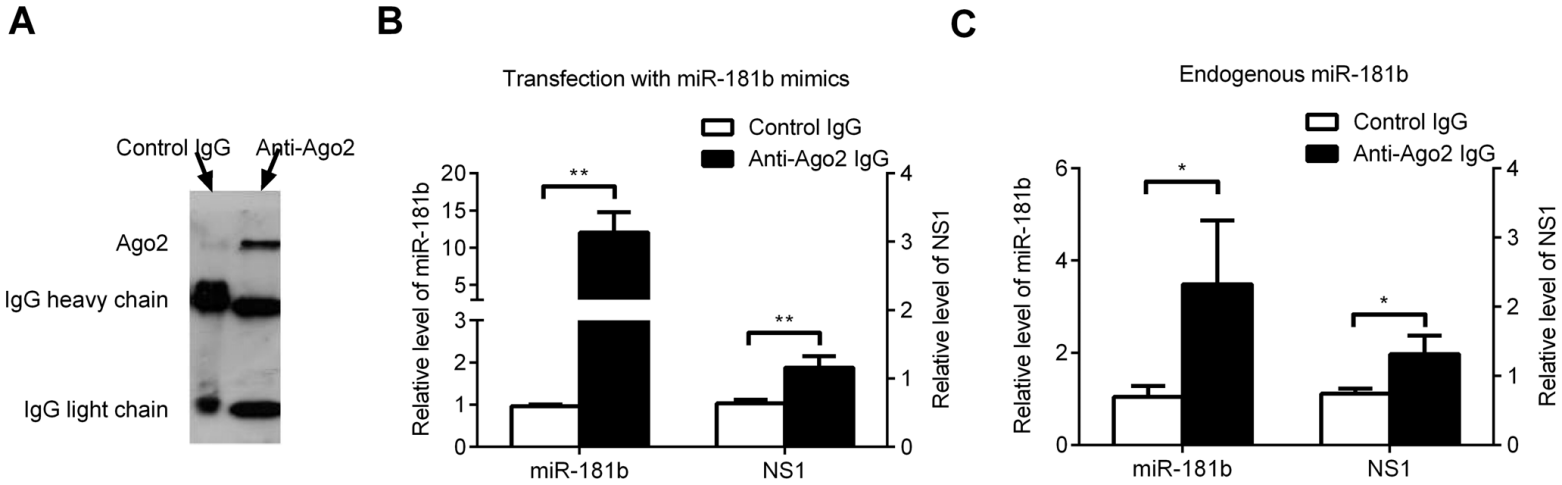
miR-181b physically binds to MEV NS1 mRNA in the RISC. (**A**) Western blot assay was used to detect Ago2 protein from Ago2 immunoprecipitates of lysates from F81 cells. (**B**) qPCR analysis of the relative level of miR-181b and MEV NS1 mRNAs from Ago2 or IgG immunoprecipitated lysates of F81 cells transfected with miR-181b mimics (50 pmol/well) in 6-well plates and 12 h later infected with MEV (MOI = 0.1), using U6 small RNA as an internal control. (**D**) qPCR analysis of the relative level of endogenous miR-181b and MEV NS1 mRNAs from Ago2 or IgG immunoprecipitated lysates without transfection with the mimics. Data are from 3 independent experiments (mean ± SD). Statistical significance was analyzed by Student’s *t* test; * *P*<0.05; ** *P*<0.01.

To provide evidence for the physical interaction of miR-181b with MEV NS1 mRNA, Argonaute 2 (Ago2) immunoprecipitation was performed. As previously described [40], both mRNA degradation and translational repression was dependent on RISC, in which the most important factor is Ago2 protein. Therefore, anti-Ago2 antibody was used to test if miR-181b and NS1 mRNA were enriched by Ago2. As expected, when Ago2 protein was specifically precipitated by anti-Ago2 antibody, no matter whether the cells were untreated or transfected with exogenous miR-181b mimics (Figure 6*A*), miR-181b mimics were enriched almost 15-fold and NS1 mRNA by approximately 2-fold by anti-Ago2 antibody after transfection with miR-181b mimics, as compared with IgG (Figure 6*B*). Endogenous miR-181b was also enriched more than 3-fold and NS1 mRNA by 1.8-fold (Figure 6*C*). These results confirm that both cellular endogenous miR-181b and exogenous transfected miR-181b mimics physically bind to MEV NS1 mRNA in the RISC.

### MEV Infection Regulates Cellular miRNAs Including miR-181b

**Figure 7:**
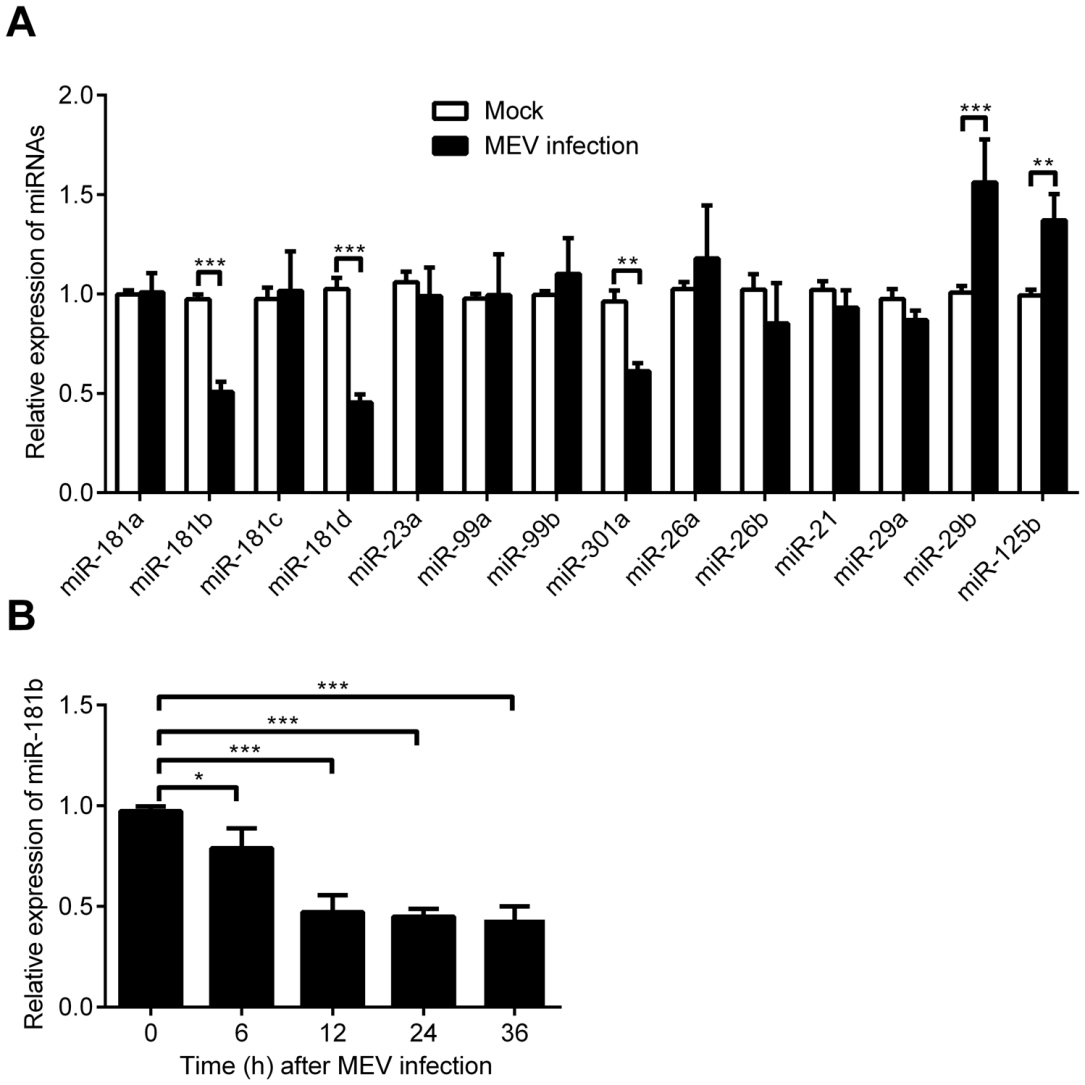
MEV infection regulates cellular miRNAs including miR-181b. (**A**) Expression of selected miRNAs in F81 cells infected with MEV (MOI = 1) and in uninfected controls were tested for validation by qPCR using a specific primer for each miRNA. Fold increase/decrease was calculated based on endogenous control U6 small RNA normalization. (**B**) qPCR was used to detect the expression levels of miR-181b at 0 h, 6 h, 12 h, 24 h and 36 h after MEV infection, normalized to U6 small RNA. Data are from 3 independent experiments (mean ± SD). Statistical significance was analyzed by Student’s *t* test; * *P*<0.05; ** *P*<0.01; *** *P*<0.001.

Since cellular miRNA inhibits MEV production as a host defence mechanism, we investigated whether MEV might modulate host miRNAs to enhance its survival. qPCR was used to test the relative expression levels of a few miRNAs (listed in Table S1) randomly selected from small RNA sequencing data (unpublished) after MEV infection. Results showed that miR-181b, miR-181d and miR-301a were significantly decreased by almost 50% after MEV infection, and miR-29b and miR-125b significantly increased by over 30% (Figure 7*A*). Although changes in the expression of most (10 of 14) of the randomly selected miRNAs except miR-99b, miR-26a, miR-21 and miR-29a were consistent with the sequencing data, the magnitude of changes was different for some miRNAs, especially miR-29b. To further confirm that miR-181b was down regulated following MEV infection, the expression of miR-181b was measured at times after MEV infection. It was found that miR-181b gradually decreased with increasing MEV infection time (Figure 7*B*). These observations demonstrate that MEV infection regulates cellular miRNAs including miR-181b.

## Discussion

Host cellular miRNAs have frequently been reported to interact with viruses during replication [20], [21], [41]–[46]. Until now, however, no such observations have been reported on single-stranded DNA parvoviruses. Here, our studies have shown that one kind of cellular miRNA, miR-181b, inhibits MEV replication by directly targeting the NS1 coding region resulting in repression of mRNA translation. As recently reported [15], [16], [47], [48], translational repression is the primary event, followed by mRNA degradation. However, according to the data in Figure 4*B*, the NS1 mRNA level was unaffected by either miR-181b mimics or inhibitors at 36 h post-transfection. The reason might be that the change of NS1 mRNA quantity level was too low to be detected.

One mechanism that viruses have evolved to circumvent host cell counteraction is by mutation resulting in multiple strains. This appeared to have occurred with MEV since many strains have been discovered [4], [49]–[53]. However, the miR-181b target site we predicted is identical in different MEV strains and even in all tested isolates of CPV and FPV (see Table 3). Being highly conserved in the targeted region among these viruses, therefore, miR-181b would appear to have an important function in CPV and FPV replication, which justifies further experimental investigation.

In our study, we found that MEV NS1 was a natural host target, perhaps because of its multiple functions: e.g., cutting the initiation site of viral DNA replication of the minute virus of mice (MVM) [56], [57] and taking part in parvovirus B19 gene expression [57]. NS1 protein exhibits ATPase, helicase, endonuclease and ATP binding activities [55], [60]. Additionally, NS1 is considered to be a tumor suppressor to promote tumor cell apoptosis [58], [59]. Parvovirus B19 NS1 specifically inhibits host heterologous DNA replication [54]. All these functions indicate that parvovirus NS1 protein is an extremely important factor not only for modulating viral replication but also for interacting with the host.

As previously reported [39], miRNAs of a single family may have the same targets. In our study, however, the relative luciferase activity of reporter vector pGL3-181t, NS1 protein expression and MEV replication was modulated by miR-181d but not miR-181c (Figure S2). The target prediction results showed that miR-181b and miR-181d had 9 sequential complementary nucleotides, whereas miR-181c had 7 (Figure 1). Although 7 nucleotides have been reported as being sufficient for inhibition of gene expression [61], it appeared that 9 were required in this case. In addition, different base pairing structures between miR-181b and miR-181c in the NS1 target sit might be related to the function of miRNAs [39]. Since miR-181b and miR-181d differ in only one nucleotide, it is possible that they have the same activity. However, following transfection with miR-181d inhibitors, elimination of the inhibitory activity of endogenous miR-181d was not detected (Figure S2 *D, E, F*). One possible reason is that the endogenous miR-181d expression level was insufficiently high (<100 copies/cell; i.e., about 10-fold lower than that of miR-181b) to have any function (Figure S1 *A*), and therefore miR-181b played the main role in MEV infection.

As summarized in Figure 7, the relative expression of some cellular miRNAs was affected by MEV infection. We surmise that since host cellular miR-181b inhibited MEV replication, in order for MEV to survive it must employ some mechanism to down regulate host negative modulator miR-181b. However, this requires further investigation.

In our preliminary investigation of the association between miR-181b and MEV *in vivo*, qPCR was used to detect the relative expression of miR-181b in different tissues of mink (Figure S3). Results showed that expression of miR-181b was lowest in intestinal tissues, especially of the large intestine. It may be that the large intestine is the organ most susceptible to infection with MEV. This is simply an inference, however, since besides targeting MEV, miR-181b has also been reported to produce degradation of some cellular targets such as PTEN, CYLD, BCL2, p27 and TIMP3 [62]–[65].

Now that miR-181b has been shown to inhibit MEV infection, it may find application as an antiviral therapeutic for MEV-induced mink enteritis. As many studies have shown [66]–[70], siRNAs can be used to treat virus diseases *in vivo*; however, little attention has so far been paid to the possibility of using endogenous miRNAs as an antivirus tool. Compared with siRNAs, endogenous miRNAs might be much safer and induce fewer side-effects. More extensive studies are merited to determine if miR-181b can be used as an antiviral tool.

To sum up, our work has shown that cellular endogenous miR-181b inhibits replication of MEV in the F81 cell line by targeting the MEV NS1 mRNA coding region resulting in mRNA translational repression, and that MEV infection also regulates cellular miRNAs. These results provide further insight into the mechanisms of MEV infection and may be useful in development of naturally-occurring miRNA antiviral strategies.

## Supporting Information

Figure S1 **Analysis of endogenous miR-181b expression and confirmation that the synthesized mimics and inhibitors function in F81 cells. (A)** Absolute qPCR analysis was performed on 4 miRNAs. **(B)** Analysis of the miR-181b target site (white box) in the 3′UTR of BCL-2 using TargetScan tools in different species including cat (*Felis domesticus*) (black box). **(C)** Relative qPCR analysis of the function of miR-181b mimics and inhibitors on the BCL-2 gene, with TfR gene as a negative control. β-actin was used as an internal control. Data are from 3 independent experiments (mean ± SD). Statistical significance was analyzed by Student’s *t* test; * *P*<0.05; ns, not significant.
(TIF)

Figure S2 **Effects of miR-181c and miR-181d on MEV replication and NS1 protein expression.** TCID_50_ values were used to quantitate the effects of miR-181c **(A)** and miR-181d **(D)** mimics and inhibitors on viral growth curves in F81 cells. NC mimics and inhibitors were used as controls. F81 cells were transfected with mimics or inhibitors for 12 h, and infected with MEV at an MOI of 0.1. Cells were collected at the indicated times and assayed. qPCR was used to assess the effects of miR-181c **(B)** and miR-181d **(E)** mimics and inhibitors of MEV genomic DNA at the indicated times from **A, D)**. The experiments were performed as in **A, D)**. Western blot assay was used to assess the effects of miR-181c **(C)** and miR-181d **(F)** mimics and inhibitors on NS1 expression. NC mimics and inhibitors were used as controls. F81 cells were co-transfected with pcDNA-NS1 together with mimics or inhibitors. The lysates of F81 cells after 36 h co-transfection were detected through western blot assay, with β-actin as an internal control. Data are from 3 independent experiments (mean ± SD). Statistical significance was analyzed by Student’s *t* test; * *P*<0.05; ** *P*<0.01; ns, not significant.
(TIF)

Figure S3 **Analysis of relative expression of miR-181b in different tissues of mink.** Analysis of the relative expression of miR-181b in mink tissues by qPCR. U6 small RNA was an internal control. Data are from 3 independent experiments (mean ± SD).
(TIF)

Table S1 **Randomly selected miRNAs expression of the two libraries.**
(TIF)
