# Supplementary figures and images for: Cellular microRNA miR-181b Inhibits Replication of Mink Enteritis Virus by Repression of Non-Structural Protein 1 Translation
Source: PLoS One. 2013 Dec 11;8(12):e81515. doi: 10.1371/journal.pone.0081515 (PMC3859502; doi:10.1371/journal.pone.0081515)

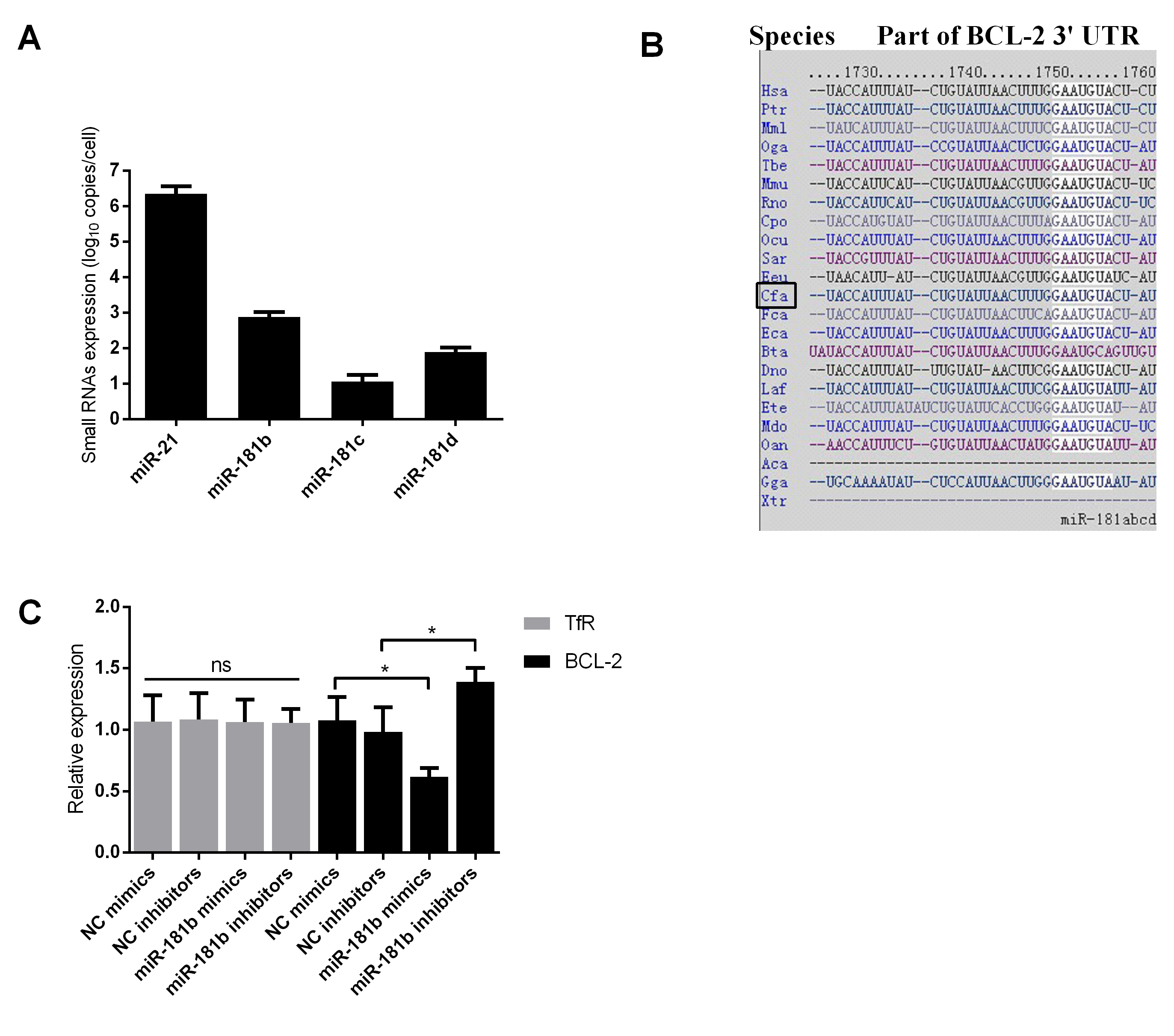

Supplement: Figure S1 — Analysis of endogenous miR-181b expression and confirmation that the synthesized mimics and inhibitors function in F81 cells. (A) Absolute qPCR analysis was performed on 4 miRNAs. (B) Analysis of the miR-181b target site (white box) in the 3′UTR of BCL-2 using TargetScan tools in different species including cat (Felis domesticus) (black box). (C) Relative qPCR analysis of the function of miR-181b mimics and inhibitors on the BCL-2 gene, with TfR gene as a negative control. β-actin was used as an internal control. Data are from 3 independent experiments (mean ± SD). Statistical significance was analyzed by Student’s t test; * P<0.05; ns, not significant. (TIF) [file pone.0081515.s001.tif]

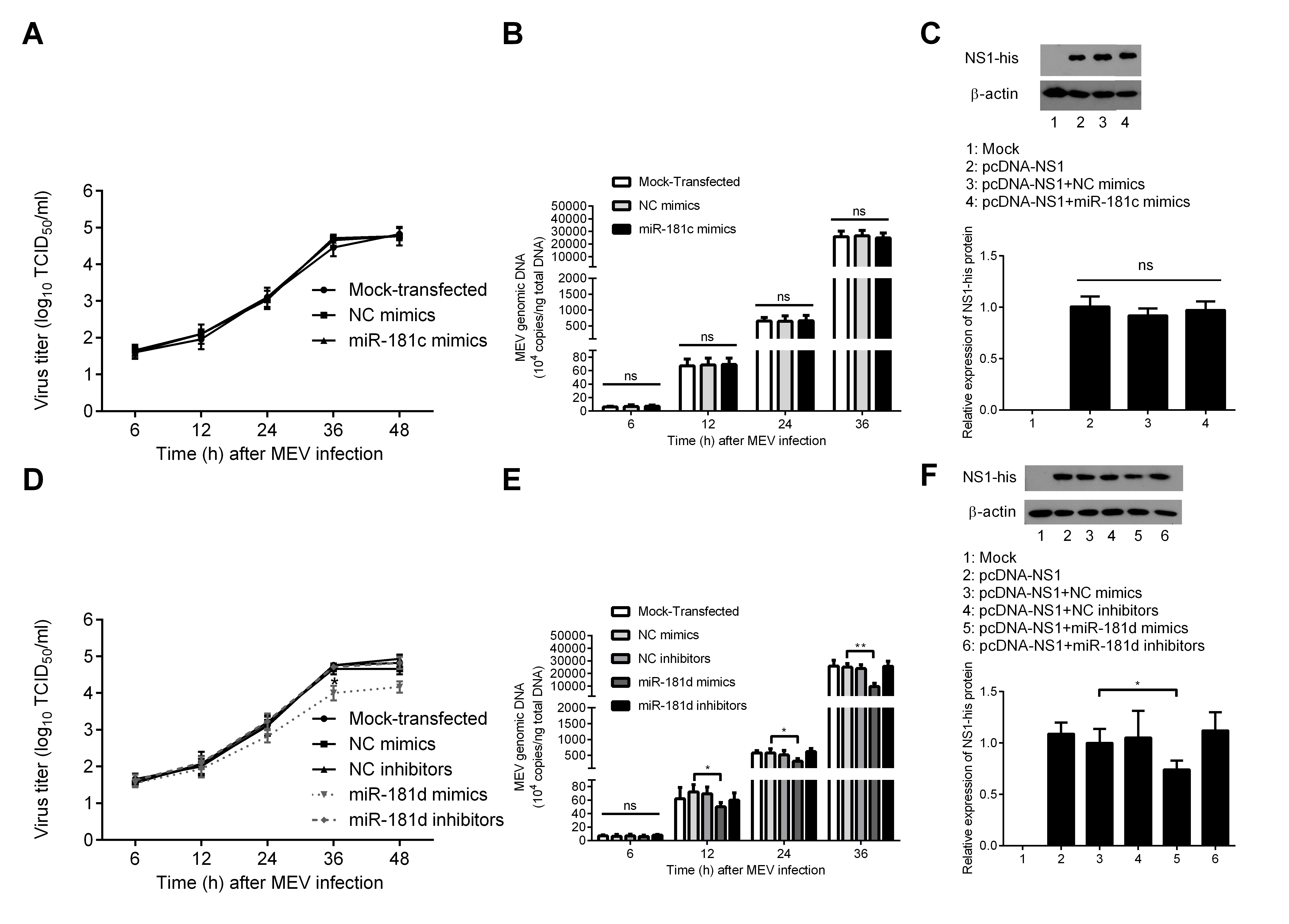

Supplement: Figure S2 — Effects of miR-181c and miR-181d on MEV replication and NS1 protein expression. TCID50 values were used to quantitate the effects of miR-181c (A) and miR-181d (D) mimics and inhibitors on viral growth curves in F81 cells. NC mimics and inhibitors were used as controls. F81 cells were transfected with mimics or inhibitors for 12 h, and infected with MEV at an MOI of 0.1. Cells were collected at the indicated times and assayed. qPCR was used to assess the effects of miR-181c (B) and miR-181d (E) mimics and inhibitors of MEV genomic DNA at the indicated times from A, D). The experiments were performed as in A, D). Western blot assay was used to assess the effects of miR-181c (C) and miR-181d (F) mimics and inhibitors on NS1 expression. NC mimics and inhibitors were used as controls. F81 cells were co-transfected with pcDNA-NS1 together with mimics or inhibitors. The lysates of F81 cells after 36 h co-transfection were detected through western blot assay, with β-actin as an internal control. Data are from 3 independent experiments (mean ± SD). Statistical significance was analyzed by Student’s t test; * P<0.05; ** P<0.01; ns, not significant. (TIF) [file pone.0081515.s002.tif]

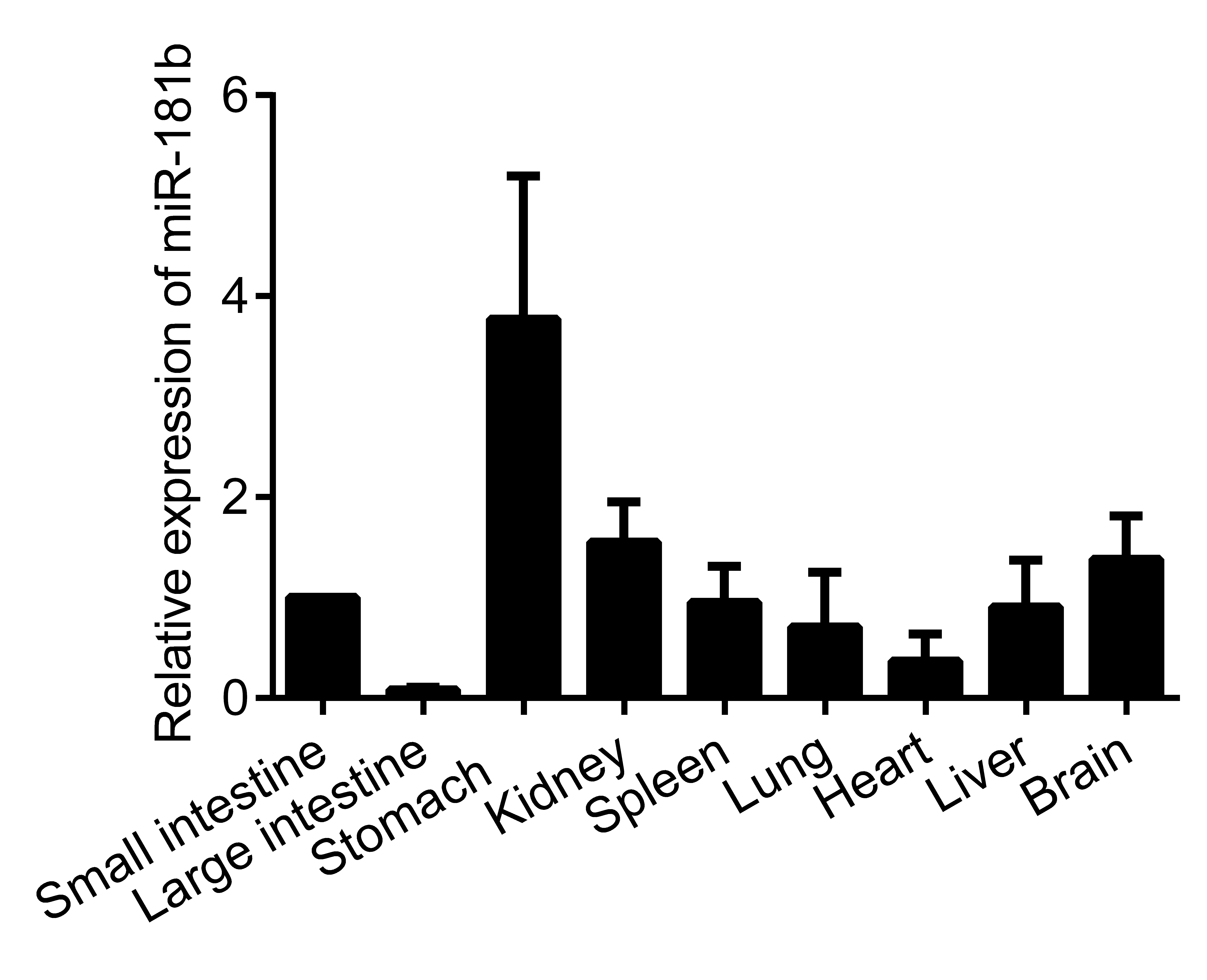

Supplement: Figure S3 — Analysis of relative expression of miR-181b in different tissues of mink. Analysis of the relative expression of miR-181b in mink tissues by qPCR. U6 small RNA was an internal control. Data are from 3 independent experiments (mean ± SD). (TIF) [file pone.0081515.s003.tif]

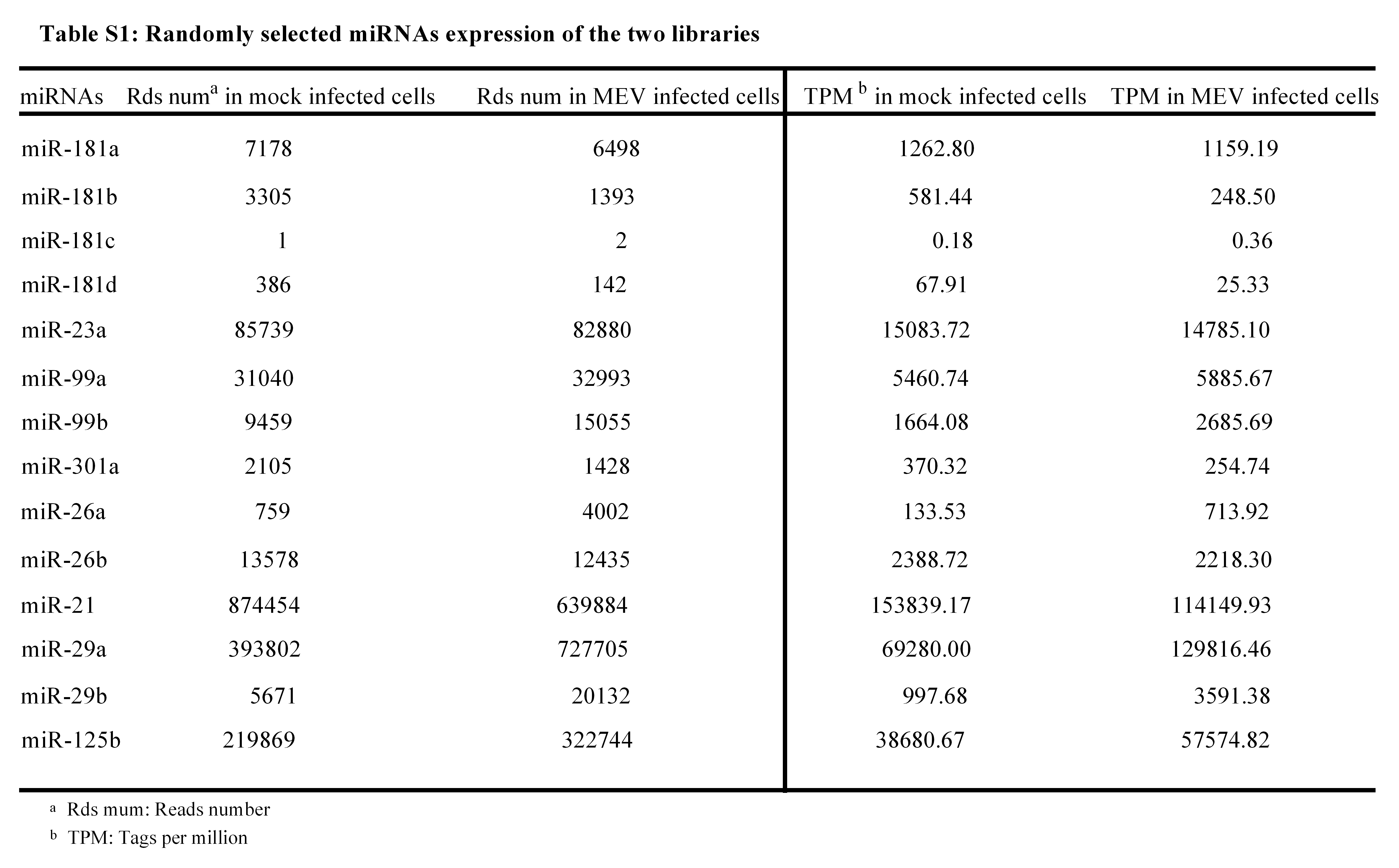

Supplement: Table S1 — Randomly selected miRNAs expression of the two libraries. (TIF) [file pone.0081515.s004.tif]
